# SPRED2 Is a Novel Regulator of Autophagy in Hepatocellular Carcinoma Cells and Normal Hepatocytes

**DOI:** 10.3390/ijms25116269

**Published:** 2024-06-06

**Authors:** Tianyi Wang, Tong Gao, Masayoshi Fujisawa, Toshiaki Ohara, Masakiyo Sakaguchi, Teizo Yoshimura, Akihiro Matsukawa

**Affiliations:** 1 Department of Pathology and Experimental Medicine, Graduate School of Medicine, Dentistry and Pharmaceutical Sciences, Okayama University, Okayama 700-8558, Japan; 2 Department of Cell Biology, Graduate School of Medicine, Dentistry and Pharmaceutical Sciences, Okayama University, Okayama 700-8558, Japan

**Keywords:** autophagy, mitophagy, SPRED proteins, MAPK/ERK, mTOR, hepatocellular carcinoma

## Abstract

Sprouty-related enabled/vasodilator-stimulated phosphoprotein homology 1 domain containing 2 (SPRED2) is an inhibitor of the mitogen-activated protein kinase (MAPK)/extracellular signal-regulated kinase (ERK) pathway and has been shown to promote autophagy in several cancers. Here, we aimed to determine whether SPRED2 plays a role in autophagy in hepatocellular carcinoma (HCC) cells. The Cancer Genome Atlas (TCGA) Liver Cancer Database showed a negative association between the level of SPRED2 and p62, a ubiquitin-binding scaffold protein that accumulates when autophagy is inhibited. Immunohistochemically, accumulation of p62 was detected in human HCC tissues with low SPRED2 expression. Overexpression of SPRED2 in HCC cells increased the number of autophagosomes and autophagic vacuoles containing damaged mitochondria, decreased p62 levels, and increased levels of light-chain-3 (LC3)-II, an autophagy marker. In contrast, SPRED2 deficiency increased p62 levels and decreased LC3-II levels. SPRED2 expression levels were negatively correlated with translocase of outer mitochondrial membrane 20 (TOM20) expression levels, suggesting its role in mitophagy. Mechanistically, SPRED2 overexpression reduced ERK activation followed by the mechanistic or mammalian target of rapamycin complex 1 (mTORC1)-mediated signaling pathway, and SPRED2 deficiency showed the opposite pattern. Finally, hepatic autophagy was impaired in the liver of SPRED2-deficient mice with hepatic lipid droplet accumulation in response to starvation. These results indicate that SPRED2 is a critical regulator of autophagy not only in HCC cells, but also in hepatocytes, and thus the manipulation of this process may provide new insights into liver pathology.

## 1. Introduction

Hepatocellular carcinoma (HCC), the primary form of liver cancer, is a major global health problem with a significant impact on morbidity and mortality. This complex malignancy often occurs in the context of chronic liver disease, which includes viral hepatitis, alcoholic liver disease, and non-alcoholic fatty liver disease [1]. Despite recent advances in medical science, HCC remains a formidable challenge that requires in-depth exploration of its underlying molecular and cellular mechanisms to develop novel approaches for its prevention and treatment.

Autophagy is a cellular process by which the body recycles its own cellular components, such as damaged proteins and organelles via the lysosomal degradation pathway [2]. This process helps maintain cellular homeostasis by removing damaged or unnecessary components and may play a role in both physiological processes, such as energy regulation and stress defense, and pathological processes, such as infection and other diseases, including cancer [3,4]. Recent studies have uncovered interesting links between Sprouty-related enabled/vasodilator-stimulated phosphoprotein homology 1 (EVH1) domain containing 2 (SPRED2) and autophagy. Overexpression of SPRED2 in HeLa or A549 cells promoted autophagosome maturation and induced autophagy-dependent cell death through direct interaction with light-chain-3 (LC3) [5]. Overexpression of SPRED2 in osteosarcoma cells also impaired cell proliferation, invasion, migration, and epithelial-mesenchymal transition (EMT) through the induction of autophagy [6].

SPRED2 is a member of the SPRED protein family and plays a critical role in cellular signaling. By acting as scaffolds, SPRED proteins, including SPRED2, inhibit the activation and intensity of extracellular signal-regulated kinase (ERK) signaling pathways [7]. We and others previously reported that the expression level of SPRED2 is reduced in HCC [8,9] and that the loss of endogenous SPRED2 leads to increased EMT and stemness in HCC cells by activating ERK and its downstream signaling pathways [8], suggesting the importance of SPRED2-mediated regulation of the mitogen-activated protein kinase (MAPK)/ERK signaling in a variety of cellular processes in HCC cells. The phosphoinositide 3-kinase (PI3K)/protein kinase b (AKT)/mechanistic or mammalian target of rapamycin complex 1 (mTORC1) pathway is the best-known regulator of autophagy, but the MAPK pathway is also involved in the regulation of this process [10,11]. Therefore, it is possible that SPRED2 regulates autophagy through regulation of the MAPK signaling pathway [12,13]; however, the involvement of the MAPK/ERK pathway in the autophagic flux in HCC cells and the role of SPRED2 in normal hepatocyte physiology remain to be elucidated.

In the present study, we aimed to define the intricate relationships between SPRED2 and autophagy in HCC cells and found that SPRED2 deficiency activates autophagic activity in HCC cells via the ERK/mTORC1 pathway. We then investigated the role of SPRED2 in autophagy of normal hepatocytes by starvation. Interestingly, autophagy was significantly downregulated in the liver of SPRED2-knockout (KO) mice under both fed and fasted conditions, as compared to wild-type (WT) mice. Thus, SPRED2 is an important regulator of autophagy not only in HCC cells, but also in normal hepatocytes. By elucidating the molecular and cellular mechanisms involved, we have provided new insights into liver pathology.

## 2. Results

### 2.1. SPRED2 Expression Level Is Negatively Associated with p62 Expression Level in HCC 

**Figure 1:**
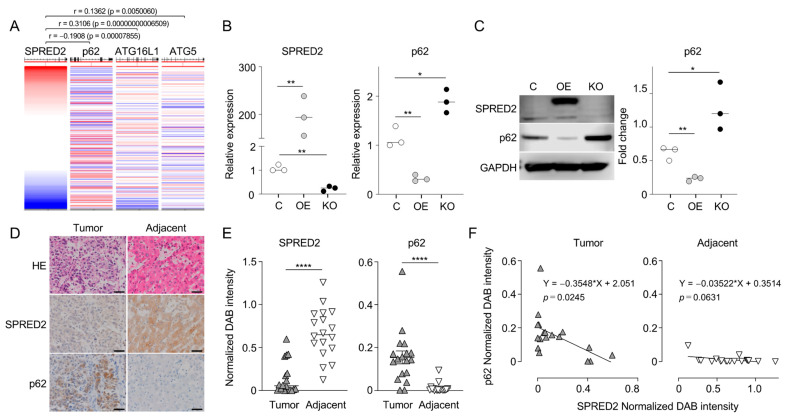
Association of SPRED2 expression with the expression of autophagy-related genes. (**A**) The relationship between SPRED2 and autophagy-related gene expression was analyzed using the data (accessed on 31 March 2023) at https://xena.ucsc.edu/ Colored red to blue for high to low expression. Lines in p62, ATG16L1, and ATG5 columns indicate expression levels in individual samples (**B**,**C**) The mRNA and protein levels of p62 in control [C], SPRED2-OE [OE], and SPRED2-KO [KO] HepG2 cells were measured by RT-qPCR (**B**) and Western blotting (**C**), respectively. Data are presented as the mean ± SEM. * *p* < 0.05, ** *p* < 0.01. n = 3. (**D**–**F**) HCC tumors and adjacent liver tissues from 18 human HCC cases were stained by HE or by IHC using anti-SPRED2 or p62 antibody. Three consecutive paraffin sections were used. (**D**) Representative images are presented. Scale bars are 50 μm. (**E**) Semi-quantitative determination of SPRED2 (left) and p62 (right) staining in the tumor and adjacent area is expressed as normalized DAB intensity. **** *p* < 0.0001. (**F**) The relationship between DAB intensity of SPRED2 and p62 in the tumor (left) and adjacent area (right) is shown.

To investigate a potential association between SPRED2 and autophagy in HCC, we initiated our analysis using data from The Cancer Genome Atlas (TCGA)–Liver Hepatocellular Carcinoma (LIHC) database. This initial analysis allowed us to assess the putative relationship between endogenous SPRED2 and genes closely associated with the autophagy process, collectively referred to as autophagy-related genes (ATGs). The results of database analyses revealed statistically significant positive associations of SPRED2 with key ATGs, such as ATG16L1 and ATG5, and a negative association with p62/sequestosome 1 (SQSTM1) (hereafter referred to as p62) (Figure 1A). p62 is a ubiquitin-binding scaffold protein, binds directly to LC3, and is degraded by autophagy. Since p62 protein levels are modulated by autophagy, p62 expression can be used as a marker to study autophagic flux [14]. Recently, we reported that the expression of SPRED2, an endogenous MAPK/ERK inhibitor, was downregulated in human HCC [8] and the transcription of p62 is regulated by the MAPK/ERK pathway [15]; therefore, the negative association of SPRED2 with p62 was of particular interest and served as the basis for our investigation of the intricate interactions and potential functional implications of SPRED2 within the autophagy pathway. 

We investigated whether SPRED2 affects p62 expression in SPRED2-KO [8] or overexpressing (OE) HepG2 cells, a human HCC cell line. The mRNA and protein levels of p62 were significantly downregulated in SPRED2-OE cells and conversely upregulated in SPRED2-KO cells, compared to control cells, via RT-qPCR (Figure 1B) and Western blotting (Figure 1C), respectively. To validate in clinical cases, we compared SPRED2 and p62 protein expression in 18 tissues from clinical HCC patients (Table 1) by immunohistochemistry (IHC) (Figure 1D). Tumor cells showed lower levels of SPRED2 immunoreactivity compared to adjacent non-tumor cells (Figure 1E, left). In contrast, p62 immunoreactivity was higher in tumor cells (Figure 1E, right). There was a negative correlation between SPRED2 and p62 in the tumor, but not in the adjacent area (Figure 1F). These results further support the hypothesis that decreased SPRED2 expression in HCC leads to the inhibition of autophagy. 

### 2.2. SPRED2 Promotes Autophagy in HCC Cells

**Figure 2:**
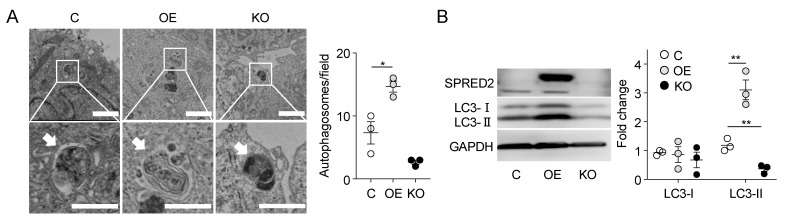
SPRED2 promotes autophagy in HCC cells. (**A**) TEM showing the presence of autophagosomes in control [C], SPRED2-OE [OE], and SPRED2-KO [KO] HepG2 cells. The area enclosed by the square is enlarged below. Arrows indicate autophagosomes. Scale bars indicate 2 μm (top) and 1 μm (bottom). The number of autophagosomes was counted in 135 μm^2^. Data are presented as the mean ± SEM. * *p* < 0.05, n = 3. (**B**) Cell lysates were prepared from control [C], SPRED2-OE [OE], and SPRED2-KO [KO] HepG2 cells and the presence of each protein was evaluated by Western blotting. Band densities were digitized and semi-quantitated. Data are presented as the mean ± SEM. ** *p* < 0.05. n = 3.

To obtain evidence that endogenous SPRED2 exerts its regulatory influence on autophagic activity in HCC, we used SPRED2-OE cells and SPRED2-KO-HepG2 cells. Cell samples were examined by transmission electron microscopy (TEM). During the autophagy process, membrane structures (autophagosomes) are formed in a single cell to deliver the engulfed substrates to lysosomes for degradation [17]. As shown in Figure 2A, a significant increase in the number of autophagosomes was observed in SPRED2-OE cells. In contrast, a reduction in the number of autophagosomes was observed in SPRED2-KO cells, although it was not statistically significant (*p* = 0.06) (Figure 2A). These results provide compelling evidence for the ability of endogenous SPRED2 to stimulate the formation of autophagosomes, resulting in an elevated state of autophagic activity.

To further demonstrate the role of endogenous SPRED2 in promoting autophagy, we examined the level of microtubule-associated LC3 by Western blotting. During autophagy, microtubule-associated LC3 is localized to autophagosomes (called LC3 puncta). LC3-I is then converted to LC3-II, which is essential for autophagosome formation [18]. As shown in Figure 2B, SPRED2-OE cells showed a significant increase in LC3-II levels compared to the control, accompanied by a marked reduction in p62 accumulation. Conversely, the level of LC3-II was significantly decreased in SPRED2-KO cells, accompanied by an accumulation of p62 (Figure 2B). 

In addition to HepG2 cells, two other HCC cell lines (Hep3B and HLE cells) were also examined. In contrast to SPRED2-KO HepG2 cells, SPRED2 downregulation by transfection with SPRED2-specific siRNA failed to alter the levels of p62 and LC3-II, probably due to the low SPRED2 levels in these two cell lines [8]. However, similar results were obtained by overexpressing SPRED2 in Hep3B or HLE cells (Figure S1). Taken together, these results suggest a regulatory role of SPRED2 in modulating p62 and LC3 dynamics, highlighting its influence on autophagic processes and further supporting the hypothesis that the reduced endogenous SPRED2 expression observed in HCC cells leads to the downregulation of autophagy.

### 2.3. Overexpression of SPRED2 Induces Mitophagy in HCC Cells

**Figure 3:**
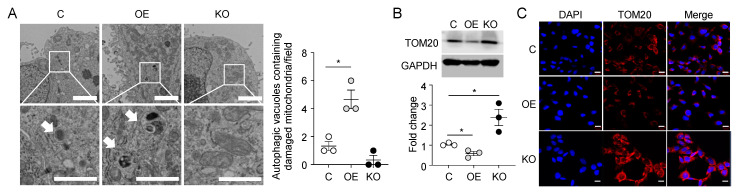
SPRED2 promotes mitophagy in HCC cells. (**A**) TEM showing the presence of autophagic vacuoles housing damaged mitochondria in control [C], SPRED2-OE [OE], and SPRED2-KO [KO] HepG2 cells. The area enclosed by the square is enlarged below. Arrows indicate autophagic vacuoles containing damaged mitochondria. Scale bars indicate 2 μm (top) and 1 μm (bottom). The number of autophagic vacuoles containing damaged mitochondria was counted in 135 μm^2^. Data are presented as the mean ± SEM. * *p* < 0.05. n = 3. (**B**) Cell lysates were prepared from control [C], SPRED2-OE [OE], and SPRED2-KO [KO] HepG2 cells and the presence of each protein was evaluated by Western blotting. Band densities were digitized and semi-quantitated. Data are presented as the mean ± SEM. * *p* < 0.05. n = 3. (**C**) Control [C], SPRED2-OE [OE], and SPRED2-KO [KO] HepG2 cells were seeded on a Lab-Tek II slide and fixed in acetone, and the presence of TOM20 was analyzed by immunofluorescence. Representative images showing TOM20 expression are shown. Blue: DAPI, Red: TOM20. Scale bars are 20 µm.

Given the relevance of SPRED2 in autophagy, we focused on determining the potential regulatory impact of SPRED2 on mitophagy. We examined the presence of autophagic vacuoles in HepG2 cells, which contain damaged mitochondria and serve as an indicator of mitophagic activity. TEM revealed a significant increase in the abundance of autophagic vacuoles containing damaged mitochondria in SPRED2-OE cells. Conversely, the number of damaged mitochondria was decreased in SPRED2-KO cells, although this was not statistically significant (*p* = 0.10) (Figure 3A). We also evaluated the level of translocase of outer mitochondrial membrane 20 (TOM20) as a selected marker to assess the level of mitochondrial autophagic activity [19]. The level of TOM20 was significantly decreased in SPRED2-OE cells, and was significantly increased in SPRED2-KO cells, compared to the control (Figure 3B). Consistent with the Western blotting results, the immunofluorescence level of TOM20 was significantly decreased in SPRED2-OE cells and increased in SPRED2-KO cells compared to the control (Figure 3C). The above changes observed upon SPRED2 overexpression in HepG2 cells were also observed in the other two cell types, Hep3B and HLE (Figure S2). These results provide a picture of the role of SPRED2 in potentiating mitophagy processes in HCC cells.

### 2.4. ERK-mTORC1-Mediated Signaling Pathway Is Inhibited by SPRED2

**Figure 4:**
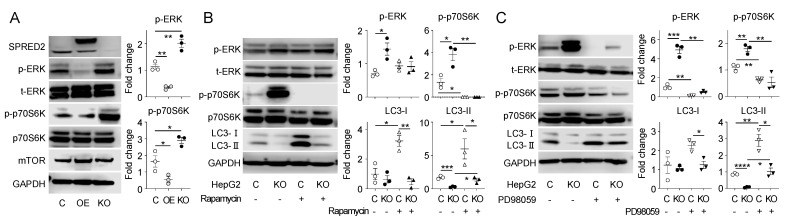
SPRED2 regulates autophagy through the ERK-mTORC1 signaling pathway in HCC cells. (**A**) Cell extracts were prepared from control [C], SPRED2-OE [OE], and SPRED2-KO [KO] HepG2 cells, and the presence of each protein was evaluated by Western blotting. Band densities were digitized and semi-quantitated. Data are presented as the mean ± SEM. * *p* < 0.05, ** *p* < 0.01. n = 3. (**B**) Control [C] and SPRED2-KO [KO] HepG2 cells were treated with 20 μm rapamycin or vehicle (DMSO) for 6 h, after which cell lysates were prepared, and the presence of each protein was assessed by Western blotting. Band densities were digitized and semi-quantitated. Data are presented as the mean ± SEM. * *p* < 0.05, ** *p* < 0.01, *** *p* < 0.001. n = 3. (**C**) Control [C] and SPRED2-KO [KO] HepG2 cells were treated with 20 μm PD98059 or vehicle (DMSO) for 1 h, after which cell lysates were prepared, and the presence of each protein was assessed by Western blotting. Band densities were digitized and semi-quantitated. Data are presented as the mean ± SEM. * *p* < 0.05, ** *p* < 0.01, *** *p* < 0.001, **** *p* < 0.0001. n = 3.

To elucidate the underlying molecular mechanisms of SPRED2-mediated regulation of autophagy in HCC cells, we investigated whether the activation of mTORC1, a core protein in the autophagy-related pathway [13], is regulated by SPRED2. We previously showed that ERK phosphorylation is decreased in SPRED2-OE cells and increased in SPRED2-KO cells [8]. Activation of mTORC1 promotes the phosphorylation of p70 S6 kinase (p70S6K), which serves as a marker for mTORC1 activation [20]. As shown in Figure 4A, the levels of p-ERK and phospho-p70S6K (p-p70S6K) were decreased in SPRED2-OE cells, whereas they were increased in SPRED2-KO cells. Overexpression of SPRED2 in Hep3B or HLE cells also decreased the level of p-p70S6K (Figure S3). These results indicate that endogenous SPRED2 inhibited the mTORC1 signaling pathway. 

To confirm the involvement of mTORC1 in autophagy of HCC cells, we used rapamycin to inhibit the mTORC1 pathway and found that inhibition of the mTORC1 pathway completely decreased the phosphorylation of p70SK6 and increased the level of LC3-II in both control cells and SPRED2-KO HepG2 cells (Figure 4B). Interestingly, the phosphorylation of ERK was unchanged by rapamycin in both cell types, suggesting that the ERK pathway is unlikely to be the downstream pathway of mTORC1 activation. To determine whether the ERK pathway regulates SPRED2-mediated mTORC1 activation, we treated HepG2 cells with the MEK inhibitor PD98509. Inhibition of ERK decreased the phosphorylation of p70SK6 and increased the level of LC3-II in both control and KO cells (Figure 4C). Taken together, these results suggest that SPRED2 regulates autophagy in HCC cells through ERK and subsequent activation of mTORC1.

### 2.5. Loss of SPRED2 Reduces Autophagy, including Mitophagy, In Vivo

**Figure 5:**
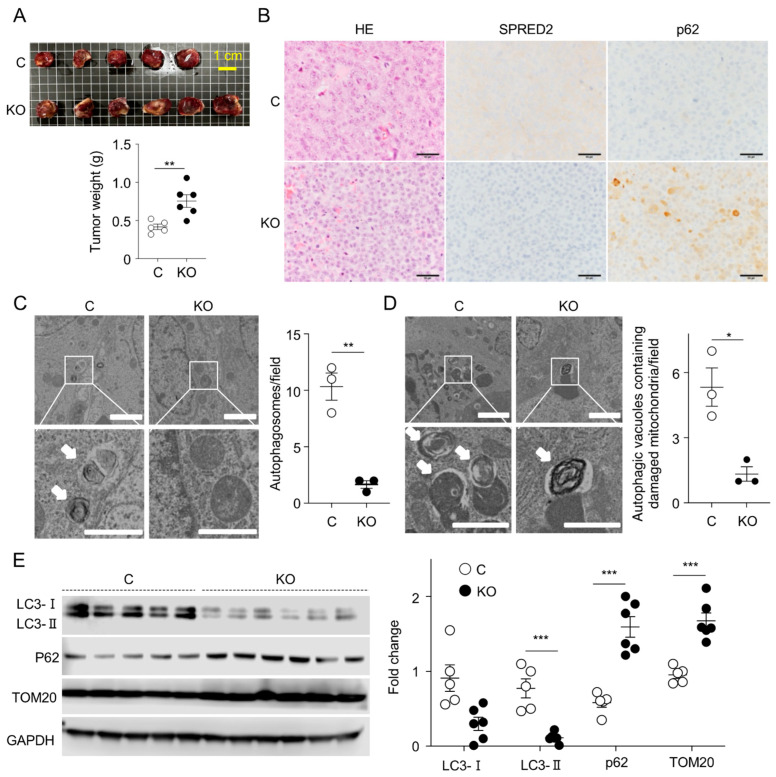
SPRED2 deficiency promotes the growth of HepG2 cells *in vivo*. (**A**–**E**) Control [C] or SPRED2-KO [KO] HepG2 cells (5 × 10^6^ cells) were subcutaneously inoculated into SCID-beige mice. Six weeks later, mice were euthanized and tumors were resected. (**A**) Upper: A photograph of the resected tumors is shown. Scale bar is 1 cm. Lower: Tumor weights were recorded, and data were analyzed. Data are presented as the mean ± SEM. ** *p* < 0.01. Control, n = 5, SPRED2-KO, n = 6. (**B**) Representative images of HE staining and immunostaining for SPRED2 and p62 are shown. Scale bars are 50 μm. (**C**) TEM showing autophagosome in tumor cells. The area enclosed by the square is enlarged below. Arrows indicate autophagosomes. Scale bars indicate 2 μm (top) and 1 μm (bottom). The number of autophagosomes was counted in 135 μm^2^. Data are presented as the mean ± SEM. ** *p* < 0.01. n = 3. (**D**) TEM showing autophagic vacuoles containing damaged mitochondria in tumor cells. The area enclosed by the square is enlarged below. Arrows indicate autophagic vacuoles containing damaged mitochondria. Scale bars indicate 2 μm (top) and 1 μm (bottom). The number of autophagic vacuoles containing damaged mitochondria was counted in 135 μm^2^. Data are presented as the mean ± SEM. * *p* < 0.05. n = 3. (**E**) Cell lysates were prepared from control [C] and SPRED2-KO [KO] tumors, and the presence of each protein was evaluated by Western blotting. Band densities were digitized and semi-quantitated. Data are presented as the mean ± SEM. *** *p* < 0.001, Control, n = 5., SPRED2-KO, n = 6.

The results obtained *in vitro* led us to investigate whether the loss of SPRED2 promotes HCC development *in vivo*. We subcutaneously inoculated control cells or SPRED2-KO-HepG2 cells into severe combined immunodeficiency (SCID)-beige mice. Six weeks later, the mice were euthanized, and the tumors were resected. Five out of six mice in the control group developed tumors, while all six mice in the KO group developed tumors. The size and weight of the SPRED2-KO tumors were larger than those of the control tumors (Figure 5A). Immunohistochemically, p62 staining was significantly higher in SPRED2-KO tumors compared to control tumors (Figure 5B). TEM was performed to compare the number of autophagosomes in the tumor samples. Fewer autophagosomes and fewer autophagic vacuoles containing damaged mitochondria were detected in SPRED2-KO tumors (Figure 5C,D). LC3-II levels were significantly lower in SPRED2-KO tumors as compared to control tumors, and tumor p62 and TOM20 levels were significantly higher in SPRED2-KO tumors compared to control tumors (Figure 5E). These results suggest that loss of SPRED2 may contribute to the increased development of HCC by inhibiting autophagy, including mitophagy.

### 2.6. Loss of SPRED2 Impairs Autophagy in Normal Hepatocytes 

**Figure 6:**
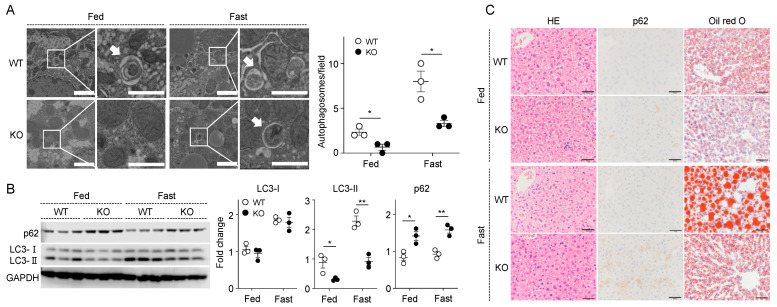
SPRED2 deficiency inhibits starvation-induced autophagy in the liver. (**A**–**C**) WT and SPRED2-KO [KO] mice were fasted for 48 h. Mice were then sacrificed, and the livers were resected. (**A**) Left: TEM showing the presence of autophagosomes in the WT or SPRED2-KO [KO] liver. The area enclosed by the square is enlarged right. Arrows indicate autophagosomes. Scale bars indicate 2 μm (left) and 1 μm (right). Right: The number of autophagosomes was counted in 135 μm^2^. Data are presented as the mean ± SEM. * *p* < 0.05. n = 3. (**B**) Left: Tissue lysates were prepared from WT and SPRED2-KO [KO] livers and the presence of each protein was assessed by Western blotting. Right: Band densities were digitized and semi-quantitated. Data are presented as the mean ± SEM. * *p* < 0.05, ** *p* < 0.01. n = 3. (**C**) Representative images of HE staining, p62 immunostaining, and Oil red O staining are shown. Scale bars are 50 μm.

To extend our efforts to define the role of SPRED2 in autophagy, we next investigated the regulatory role of SPRED2 in autophagy of normal hepatocytes. Starvation is a potent physiological inducer of autophagy in the liver, and a quick and easy way to stimulate the autophagy machinery in mice [21,22]. We fasted WT and Spred2-KO mice for 48 h and compared the level of autophagy in the liver of mice that were continued to be fed. Using TEM, a small number of autophagosomes were found in WT liver, but they were barely detectable in SPRED2-KO liver under fed conditions. The number of autophagosomes was significantly lower in SPRED2-KO liver than in WT liver under both conditions (Figure 6A). Via Western blotting, there was no difference in the level of LC3-I between WT and SPRED2-KO liver under both conditions. LC3-II levels were higher in WT than in SPRED2-KO liver, and conversely, p62 levels were significantly higher in SPRED2-KO than in WT liver under both conditions (Figure 6B). Under fed conditions, the intensity of p62 staining was slightly higher in SPRED2-KO liver than in WT liver. Oil red O staining showed that SPRED2-KO mice, like other autophagy-deficient mice [23,24], had impaired hepatic accumulation of lipid droplets in response to fasting (Figure 6C). These results suggest that endogenous hepatic SPRED2 is an important molecule to protect the liver from potential injury caused by physiological stimuli, such as starvation, by regulating autophagic flux.

## 3. Discussion

There is increasing evidence that autophagy plays a critical role in regulating liver physiology and balancing hepatic metabolism. Conversely, autophagy can also contribute to the onset and development of liver diseases, such as hepatic steatosis, hepatitis, fibrosis, cirrhosis, and HCC. In one aspect, autophagy protects hepatocytes from damage and cell death by eliminating damaged organelles and proteins introduced in liver-related diseases [25]. Therefore, the proper regulation of autophagy in different situations is very important for the prevention and treatment of liver injury. 

In the present study, we aimed to determine the role of SPRED2 in autophagy of HCC cells, the potential molecular mechanisms behind SPRED2, and its association with the suppression of HCC development. Overexpression of SPRED2 in HCC cell lines resulted in increased autophagy and mitophagy, whereas deletion of SPRED2 resulted in a decrease via the ERK-mTORC1 signaling pathway. SPRED2-KO cells formed significantly larger tumors with decreased autophagic activities *in vivo*. Finally, we demonstrated, for the first time, that starvation-induced autophagy of normal hepatocytes is reduced in SPRED2-KO mice. These results indicate that SPRED2 is critical for the regulation of autophagic activities not only in HCC cells, but also in normal hepatocytes, and suggest its role in the development of liver diseases, including HCC. SPRED2 inhibits Ras-dependent ERK1/2 signaling by suppressing the phosphorylation and activation of Raf through interaction with Ras [7]. ERK has been shown to phosphorylate Raptor, a protein in the mTORC1 complex [26]. Thus, reduced SPRED2 expression results in upregulation of ERK and mTORC1. We recently showed that downregulation of SPRED2 in HCC promotes tumor cell proliferation, EMT, stemness, and anti-apoptotic properties, and leads to more malignant phenotypes through increased activation of MAPK/ERK [8]. We and others have shown that SPRED2 expression is downregulated in many cancers, including HCC [6,8,9,27,28,29]. Autophagy serves as an important cytoprotective process by maintaining cellular homeostasis and recycling cytoplasmic contents; however, recent evidence suggests that autophagy is a primary mechanism of cell death [30]. Our present data suggest that reduced SPRED2 expression in cancer may contribute to cancer proliferation and progression, at least in part, by inhibiting autophagic activities. 

mTORC1 plays a key role in the regulation of autophagy [13]. Our study showed that SPRED2 promotes autophagy by inhibiting mTORC1 in a MAPK/ERK-dependent manner. SPRED2 inhibits the activation of MAPK/ERK by interacting with rat sarcoma (RAS) [7], ribosomal S6 kinase (RSK) 1, neurofibromin [31], and RSK2 [32]. SPRED2 appears to activate autophagy by interacting with selective autophagic components, such as LC3 [5], p62, a neighbor of breast cancer susceptibility gene I (BRCA1) and cathepsin D [33], as evidenced by co-immunoprecipitation of these molecules with SPRED2. Although we did not examine the interaction between SPRED2 and autophagic components in HCC cells, our finding that the inhibition of MAPK/ERK by PD98059 inhibited the activation of mTORC1 and subsequently activated autophagy in the absence of SPRED2 in SPRED2-KO cells strongly suggests the importance of MAPK/ERK in SPRED2-mediated autophagy in HCC cells. Interestingly, inhibition of MAPK signaling by the MEK1/2 inhibitor selumetinib led to an increase in autophagic flux in cardiac cells [33]. An important role of the MAPK-mTORC1 pathway in autophagy is further supported by the finding that activation of the MAPK pathway leads to the phosphorylation of Raptor, an essential scaffolding protein of mTORC1 [26]. Inhibition of mitogen-activated protein kinase kinase 1/2 (MEK1/2) inhibition also led to activation of the liver kinase B1/adenosine monophosphate (AMP)-activated protein kinase/Unc-51-like kinase 1 signaling axis, a key regulator of autophagy, in pancreatic cancer cells [10]. Furthermore, ERK-dependent activation of mTORC1 has been shown to be mediated by phosphorylation of tuberous sclerosis 2 (TSC2) and its downstream target Ras homolog enriched in brain (Rheb) in myoblasts [34]. Therefore, the interaction between SPRED2 and molecules involved in autophagy signaling needs to be elucidated. 

The PI3K/AKT/mTORC pathway is a well-known canonical autophagy-inducing pathway [35]. It has been suggested that the MAPK/ERK and the PI3K/AKT pathways cross-regulate each other and co-regulate downstream functions [11,35]. To address this, we examined the interaction of these two pathways. There was no change in AKT activation in SPRED2-OE or SPRED2-KO cells, as compared to the control (Figure S4A). PI3K inhibitor 3-Methyladenine (3-MA) reduced AKT activation without altering ERK activation (Figure S4B). Thus, these pathways appear to be independent of each other. 

Starvation induces autophagy in several organs in mice, but a strong induction was detected in the heart, skeletal muscle, and liver [21,22]. An indispensable role of SPRED2 in the regulation of cardiac autophagy has been shown previously [33,36]. We showed that autophagy was significantly reduced in hepatocytes of both fed and fasted SPRED2-KO mice, suggesting a critical role of SPRED2 in liver physiology by regulating autophagic flux. It will be interesting to see whether SPRED2 is also involved in the regulation of autophagy in other organs during starvation. 

In conclusion, we have provided new evidence strongly suggesting that SPRED2 functions as a critical regulator of autophagy in the liver. The results of this study, together with our previous finding that there is a negative correlation between SPRED2 expression levels and cancer grades in HCC, further support the hypotheses that a reduction in or loss of SPRED2 expression in hepatocytes contributes to the initiation and development of HCC, and that SPRED2 serves as a potential biomarker for HCC.

## 4. Materials and Methods

### 4.1. Data Collection

mRNA expression profiles of SPRED2 genes and autophagy-related genes (ATGs) in a total of 438 HCC cases (obtained by RNAseq) were collected from the TCGA–Liver Hepatocellular Carcinoma (LIHC) database (accessed on 31 March 2023) using the University of California, Santa Cruz (UCSC) Xena Browser (https://xena.ucsc.edu/). Expression heatmaps of defined gene sets were then generated and clustered online.

### 4.2. Human Tissue Samples

A total of 18 surgically resected HCC specimens (Table 1) were retrieved from the pathology records of Okayama University Hospital. The patients who received chemotherapy or radiotherapy before the resection were not included in this study. Two randomly selected cases were used in this study, the hematoxylin and eosin (HE)-stained sections were reviewed by two blinded pathologists, and the corresponding paraffin block was used for immunohistochemistry. The protocol of this study was reviewed and approved by the Ethics Committee of Okayama University (1703-007). Although individual written informed consent was not obtained, we disclosed the study plan on our website to allow patients or their families to opt-out, and only the cases without their refusal were enrolled in the study. Two randomly selected cases were used in this study.

### 4.3. Cell Culture 

HepG2 and HLE cells (Japanese Collection of Research Bioresources (JCRB) Cell Bank, Osaka, Japan) were cultured in Dulbecco’s modified Eagle’s medium (Nacalai Tesque, Kyoto, Japan) supplemented with 10% fetal bovine serum (FBS) (Gibco, Carlsbad, CA, USA), 100 U/mL penicillin, and 100 μg/mL streptomycin (Sigma-Aldrich, St. Louis, MO, USA). Hep3B cells (DS Pharma Biomedical, Osaka, Japan) were cultured in Eagle’s minimal essential medium (MEM) (Sigma-Aldrich) supplemented with MEM non-essential amino acid solution, 10% FBS, and antibiotics. The generation of SPRED2 KO HepG2 cells was described previously [8]. Briefly, HepG2 cells (2 × 10^5^ cells) were seeded in a 6-well plate. After overnight incubation, cells were transfected with SPRED2 Double Nickase Plasmid (sc-404738-NIC) or Control Double Nickase Plasmid (sc-437281) (Santa Cruz, Dallas, TX, USA) using Lipofectamine 3000 (Thermo Fisher, Waltham, MA, USA). The sequences for the sgRNAs used to disrupt the SPRED2 gene were 5′-GCTGATGCCCGAGCCTTTGA-2′ and 5′-GCAATCGAAGACCTTATAGA-3′. Seventy-two hours after transfection, the medium was changed to the same medium containing puromycin (2 μg/mL), and transfected cells were selected for 5 days in the presence of puromycin. Single-cell clones were then selected by serial dilution. In some experiments, cells were treated with the mTORC1 inhibitor, rapamycin (Sigma-Aldrich), for 6 h, the MEK inhibitor PD08590 (Cell Signaling Technology, Danvers, MA, USA) for 1 h, the PI3K inhibitor 3-MA (Sigma-Aldrich) for 5 h, or vehicle (Dimethyl sulfoxide: DMSO., Wako, Osaka, Japan). All experiments were performed using mycoplasma-free cells.

### 4.4. Transfection

Transfection was performed using Lipofectamine 3000 (Thermo Fisher) in OPTI-MEM 1X reduced serum medium (Gibco) for 48 h. To overexpress SPRED2 protein, HCC cells were transfected with 8 μg of the pCMViR-TSC plasmid [37] in which human SPRED2 cDNA was inserted into the EcoRI/XhoI site. The expressed recombinant SPRED2 protein contained approximately 50 additional amino acids at the end of the C-terminus. The efficacy of the overexpression was validated by real-time quantitative PCR (RT-qPCR) or Western blotting.

### 4.5. Transmission Electron Microscopy (TEM)

Sample processing included fixation with 2.5 wt% glutaraldehyde (Nacalai Tesque), staining with 2 wt% osmium (TAAB Laboratories Equipment, Aldermaston, UK), and graded dehydration in increasing concentrations of ethanol before embedding in epoxy resin (TAAB Laboratories Equipment). Ultrathin sections were cut (Leica EM UC7, Leica, Vienna, Austria) before observation by high-resolution TEM (HR-TEM, 40–120 kV) using a HITACHI TEM (model H-7650., Hitachi High-Tech Corporation, Tokyo, Japan).

### 4.6. Western Blotting

Cells were lysed in a lysis buffer (Cell Signaling Technology). Protein concentration in the lysates was measured by micro bicinchoninic acid (BCA) protein assay (TaKaRa, Kusatsu, Shiga, Japan). Equal amounts of samples (15 μg) were fractionated by sodium dodecyl sulphate-polyacrylamide gel electrophoresis (Thermo Fisher) and the proteins were transferred to polyvinylidene difluoride (PVDF) membranes. After blocking, the membranes were incubated overnight with a primary antibody, followed by a horseradish peroxidase-conjugated secondary antibody. Target proteins were visualized using ImmunoStar LD (Wako), and the membranes were scanned using a C-DiGit Blot Scanner (LI-COR Biotechnology, Lincoln, NE, USA). The blot images were semi-quantitated using Image Studio software (Ver 3.1., LI-COR Biotechnology). The antibodies used for Western blotting are listed in Table 2. 

### 4.7. Fluorescence Immunostaining

Cells were seeded on Lab-Tek II slide (8 chambers, Electron Microscopy Sciences, Hatfield, PA, USA) for 1 day at 37 °C. Cells were fixed in acetone and immunostained with the indicated primary antibodies (Table 2). Slides were then incubated with Alexa Fluor 568-conjugated anti-rabbit IgG and visualized by confocal laser scanning microscopy (LSM780, Zeiss Microscopy, Jena, Germany).

### 4.8. Immunohistochemistry (IHC) 

Tissue sections of 4 μm thickness were deparaffinized, rehydrated, treated with 0.3% H_2_O_2_ in methanol, and then subjected to heat-induced epitope retrieval in 1 mM ethylenediaminetetraacetic acid (EDTA) buffer (pH 8.0). Sections were incubated with a polyclonal anti-human SPRED2 antibody and anti-human p62 antibody (Table 2). After washing, sections were incubated with appropriate horseradish peroxidase-conjugated secondary antibody (Nichirei, Tokyo, Japan) and signals were visualized with diaminobenzidine (Dako, Santa Clara, CA, USA) according to the manufacturer’s instructions. Nuclear counterstaining was performed with hematoxylin. Semi-quantitative determination of protein expression was performed with the use of ImageJ Fiji (Ver 1.2., U. S. National Institutes of Health, Bethesda, MD, USA) according to a published protocol [38]. SPRED2 and p62 staining in the tumor and adjacent area was expressed as normalized DAB intensity [39]

### 4.9. Animals Study

Male severe combined immune deficiency (SCID)-beige mice were purchased from The Jackson Laboratory Japan (Hamamatsu, Japan). The generation of SPRED2-KO mice was described previously [7]. Male C57BL/6 mice were used as WT controls. All mice (6–8 weeks of age) were maintained in a specific pathogen-free animal facility on a 12 h light/dark cycle, at an ambient temperature of 21 °C, with ad libitum access to food and water. All animal procedures were approved by the Animal Use Committee of Okayama University Graduate School of Medicine, Dentistry and Pharmaceutical Sciences (OKU-2022618, 23866) and were performed in accordance with the guidelines of the National Institutes of Health. The endpoint of this study was when tumors reached 2 cm in diameter or when mice lost 20% of their original body weight or became inactive or moribund. 

For *in vivo* tumorigenesis assay, 5 × 10^6^ control or SPRED2-KO HepG2 cells in 100 μL Dulbecco’s modified Eagle’s medium (Nacalai Tesque, Kyoto, Japan) were injected subcutaneously into SCID-Beige mice. Six weeks later, all mice were sacrificed. Tumors were harvested, weighed, photographed, and protein extracted for Western blotting. Portions of the tumors were fixed in 10% neutral buffered formalin, embedded in paraffin, and 4 µm thick sections were stained with HE and used for IHC analysis. The other part of the tumors was fixed in 2.5 wt% glutaraldehyde and used for TEM. Each group contained at least 6 mice. 

For the *in vivo* autophagy study, WT and SPRED2-KO mice were fasted for 48 h and then sacrificed. Livers were excised and protein extracted for Western blotting. A portion of the liver was fixed in 10% neutral buffered formalin for HE staining and IHC and in 2.5 wt% glutaraldehyde for TEM. For lipid staining, a portion of liver was rapidly frozen in liquid nitrogen-cooled isopentane, and OCT compound-embedded sections were air-dried and stained with Oil red O.

### 4.10. Statistics

All statistical calculations were performed with GraphPad Prism 6 (GraphPad Software, San Diego, CA, USA). After assessing the normality of the data, statistical significance was analyzed using the parametric two-tailed unpaired t-test. Data are expressed as mean ± SEM. To evaluate the relationship between variables, linear regression analyses were used. A value of *p* < 0.05 was considered statistically significant.

## Figures and Tables

**Table 1:** Cases for the enrolled HCC patients.

Case	Age	Sex	Stage	Growth Pattern	Tumor Grade	Cirrhosis
1	56	M	1a	trabecular	moderate	-
2	79	M	1b	mixed	moderate	+
3	74	M	1a	solid	moderate	+
4	79	M	2	mixed	moderate	-
5	82	F	2	trabecular	moderate	-
6	52	M	1a	trabecular	moderate	+
7	62	M	1a	trabecular	moderate	+
8	63	M	1a	pseudoglandular	well	-
9	73	F	1b	mixed	moderate	-
10	70	M	1b	mixed	moderate	-
11	69	M	1a	trabecular	moderate	+
12	62	M	1b	trabecular	moderate	-
13	61	M	1b	mixed	moderate	-
14	69	M	1b	mixed	moderate	-
15	69	F	1a	trabecular	moderate	+
16	73	M	1b	trabecular	well	-
17	58	F	1a	trabecular	moderate	-
18	71	M	2	pseudoglandular	moderate	-

Tumor stage, tumor grade and growth pattern are classified according to the 2019 WHO classification [16]. Stage1a: Solitary tumor 2 cm or less in greatest dimension with or without vascular invasion. Stage1b: Solitary tumor more than 2 cm in greatest dimension without vascular invasion. Stage2: Solitary tumor with vascular invasion more than 2 cm dimension or multiple tumors, none more than 5 cm in greatest dimension.

**Table 2:** Antibodies for Western blotting and immunohistochemistry.

Antigens	Sources (Catalog Numbers)
SPRED2	Proteintech (24091)
LC3A/B	Cell Signaling Technology (D3U4C)
p62/SQSTM1	Cell Signaling Technology (D5L7G)
TOM20	Cell Signaling Technology (D8T4N)
P44/42 MAPK (ERK1/2)	Cell Signaling Technology (137F5)
Phospho-p44/42 MAPK (ERK1/2) (Thr202/Tyr204)	Cell Signaling Technology (D13.14.4E)
p70 S6 Kinase	Cell Signaling Technology (9202)
Phospho-p70 S6 Kinase (Thr389)	Cell Signaling Technology (108D2)
mTOR	Cell Signaling Technology (7C10)
AKT	Cell Signaling Technology (C6717)
Phospho-AKT (Ser473)	Cell Signaling Technology (S473)
GAPDH	Cell Signaling Technology (14C10)

## Data Availability

The data that support the findings of this study are available from the corresponding author upon reasonable request.

## Supplementary Materials

The following supporting information can be downloaded at: https://www.mdpi.com/article/10.3390/ijms25116269/s1.
