# Supplementary material for: SPRED2 Is a Novel Regulator of Autophagy in Hepatocellular Carcinoma Cells and Normal Hepatocytes
Source: Int J Mol Sci. 2024 Jun 6;25(11):6269. doi: 10.3390/ijms25116269 (PMC11172722; doi:10.3390/ijms25116269)
Supplement: Supplementary file 1 [file ijms-25-06269-s001.zip › ijms-3017524-supplementary.pdf]

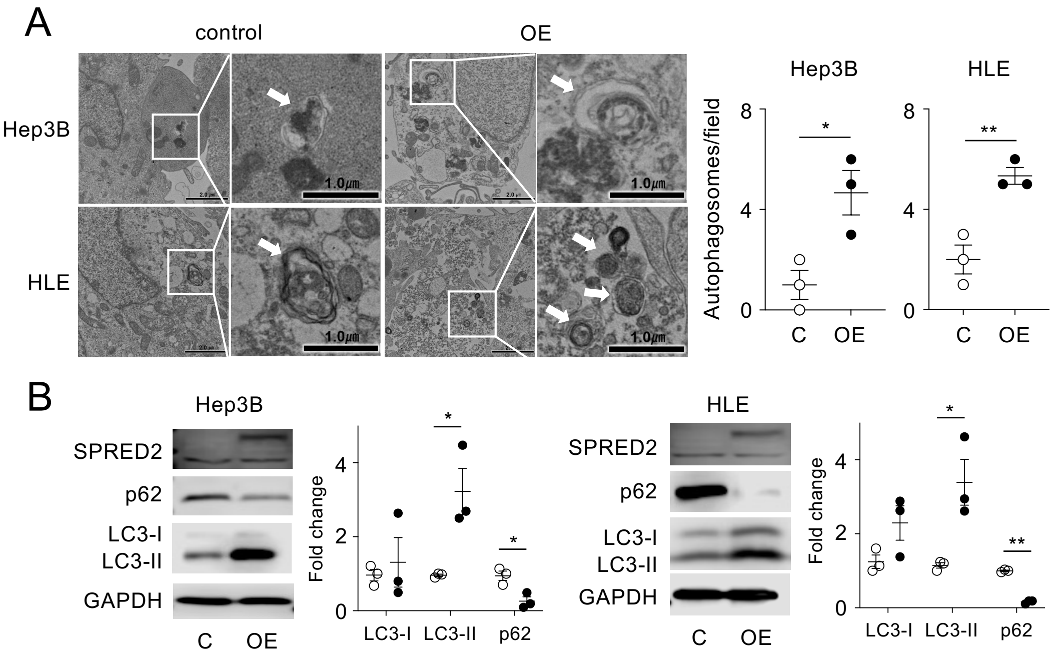

Figure S1. SPRED2 promotes autophagy in Hep3B and HLE cells. (**A**) TEM showing the presence of autophagosomes in control [C] and SPRED2-overexpressing (OE) [OE] cells. The area enclosed by the square is enlarged right. Arrows indicate autophagosomes. Scale bars are 1 μm. The number of autophagosomes was counted in 135 μm^2. Data are presented as the mean ± SEM. *$p < 0.05$, **$p < 0.01$. n = 3. (**B**) Cell lysates were prepared from control [C] and SPRED2-OE [OE] cells and the presence of each protein was evaluated by Western blotting. Band densities were digitized and semi-quantitated. Data are presented as the mean ± SEM. *$p < 0.05$, **$p < 0.01$. n = 3.

Figure S2

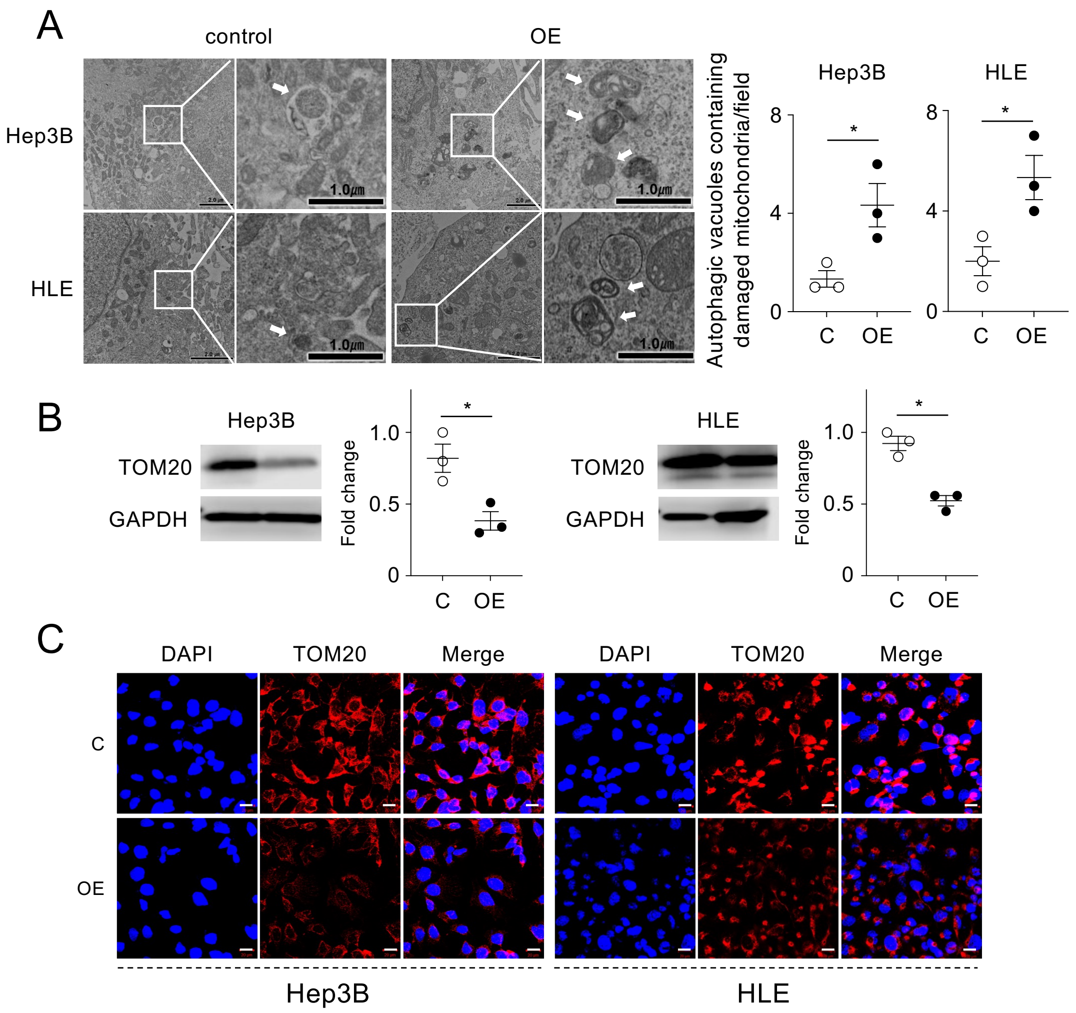

Figure S2. SPRED2 promotes mitophagy in Hep3B and HLE cells. (**A**) TEM showing the presence of autophagic vacuoles housing damaged mitochondria in control [C] and SPRED2-overexpressing [OE] cells. The area enclosed by the square is enlarged right. Arrows indicate autophagosomes. Scale bars indicate 1 µm. The number of autophagic vacuoles containing damaged mitochondria was counted in 135 μm^2. Data are presented as the mean ± SEM. *$p < 0.05$. n = 3. (**B**) Cell lysates were prepared from control [C] and SPRED2-OE [OE] cells and the presence of each protein was evaluated by Western blotting. Band densities were digitized and semi-quantitated. Data are presented as the mean ± SEM. *$p < 0.05$. n = 3. (**C**) Control [C] and SPRED2-OE [OE] cells were seeded on a Lab-Tek II slide, fixed in acetone and the presence of TOM20 was analyzed by immunofluorescence. Representative images showing TOM20 expression are shown. Blue: DAPI, Red: TOM20. Scale bars are 20 µm.

Figure S3

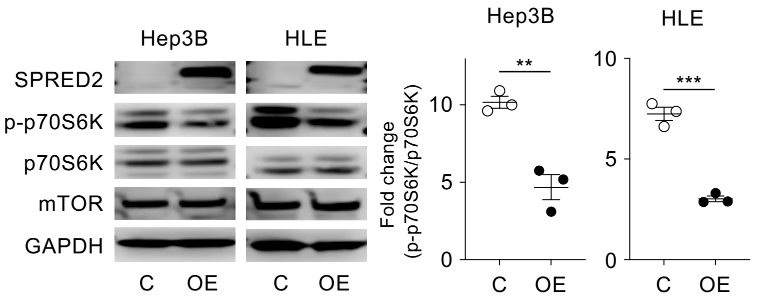

Figure S3. SPRED2 regulates autophagy through the mTORC1 signaling pathway in Hep3B and HLE cells. Cell extracts were prepared from control [C] and SPRED2-overexpressing [OE] cells, and the presence of each protein was evaluated by Western blotting. Band densities were digitized and semi-quantitated. Data are presented as the mean ± SEM. $^{**}p < 0.01$, $^{***}p < 0.001$. n = 3

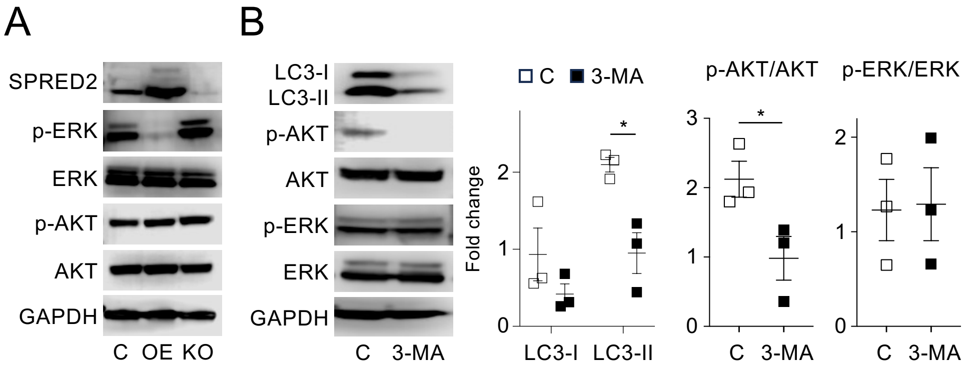

Figure S4. MAPK/ERK pathway is independent of PI3K/AKT pathway. (**A**) Cell extracts were prepared from control [C], SPRED2-overexpressing [OE] and SPRED2-kockout [KO] cells, and the presence of each protein was evaluated by Western blotting. (**B**) HepG2 cells were treated with 3 mM PI3K inhibitor 3-Methyladenine (3-MA) or vehicle control (ultra pure distilled H_2O: C) for 5 hours, after which cell lysates were prepared, and the presence of each protein was assessed by Western blotting. Band densities were digitized and semi-quantitated. Data are presented as the mean ± SEM. *$p < 0.05$. n = 3
